# Responses of non‐native and native plant species to fluctuations of water availability in a greenhouse experiment

**DOI:** 10.1002/ece3.11692

**Published:** 2024-07-09

**Authors:** Wenchao Qin, Yan Sun, Heinz Müller‐Schärer, Wei Huang

**Affiliations:** ^1^ Wuhan Botanical Garden Chinese Academy of Sciences Wuhan China; ^2^ University of Chinese Academy of Sciences Beijing China; ^3^ College of Resources and Environment Huazhong Agricultural University Wuhan China; ^4^ Department of Biology University of Fribourg Fribourg Switzerland; ^5^ Hubei Key Laboratory of Wetland Evolution & Ecological Restoration, Wuhan Botanical Garden Chinese Academy of Sciences Wuhan China

**Keywords:** biomass allocation, climate change, fluctuating resource availability hypothesis, invasive plant, soil moisture, water pulse

## Abstract

Water availability strongly influences the survival, growth, and reproduction of most terrestrial plant species. Experimental evidence has well documented the effect of changes in total amount of water availability on non‐native vs. native plants. However, little is known about how fluctuations in water availability affect these two groups, although more extreme fluctuations in water availability increasingly occur with prolonged drought and extreme precipitation events. Here, we grew seven non‐native and seven native plant species individually in the greenhouse. Then, we exposed them to four watering treatments, each treatment with the same total amount of water, but with different divisions: W1 (added water 16 times with 125 mL per time), W2 (8 times, 250 mL per time), W3 (4 times, 500 mL per time), and W4 (2 times, 1000 mL per time). We found that both non‐native and native plants produced the most biomass under medium frequency/magnitude watering treatments (W2 and W3). Interestingly, non‐native plants produced 34% more biomass with the infrequent, substantial watering treatment (W4) than with frequent, minor watering treatment (W1), whereas native plants showed opposite patterns, producing 26% more biomass with W1 than with W4. Differences in the ratio of root to shoot under few/large and many/small watering treatments of non‐native vs. native species probably contributed to their different responses in biomass production. Our results advance the current understanding of the effect of water availability on non‐native plants, which are affected not only by changes in amount of water availability but also by fluctuations in water availability. Furthermore, our results indicate that an increased few/large precipitation pattern expected under climate change conditions might further promote non‐native plant invasions. Future field experiments with multiple phylogenetically controlled pairs of non‐native and native species will be required to enhance our understanding of how water availability fluctuations impact on non‐native invasions.

## Graphical abstract

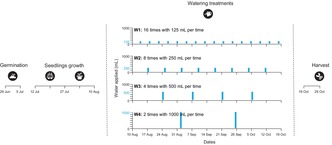

## 1 INTRODUCTION

Human activities and human‐induced climate changes are widely considered to be important drivers of non‐native plant invasions (Jauni et al., 2015; Pyšek et al., 2020; Seebens et al., 2020). This is partly because these disturbances could cause fluctuations in available resources that are not exploited by the native community, exceed its requirement, or both (Fluctuating resource availability hypothesis; Davis et al., 2000). The importance of fluctuating resource availability for non‐native plant invasions has been confirmed by a large number of experimental evidence in terms of nutrients. For example, compared to nutrients supplied constantly, multiple small pluses and a single large pulse strongly increased the ratio of non‐native plants in the native communities (Parepa et al., 2013; Tao et al., 2021) or promoted non‐native plants growth when they grew individually (Liu & van Kleunen, 2017; Tao et al., 2023). In addition to nutrients, water is vital for the survival, growth, and reproduction of terrestrial plants (Castillioni et al., 2022; Eziz et al., 2017; Mojzes et al., 2020). Given that future precipitation regimes are predicted to change drastically in most ecosystems (Bao et al., 2017; Donat et al., 2016; Pendergrass & Hartmann, 2014), such as more severe droughts and extreme precipitation, outcomes of variation in water availability on non‐native plant invasions have received growing attention (Keen et al., 2024; Koerner et al., 2015; Valliere et al., 2019).

In response to climate warming, some parts of the world are experiencing greater increases in precipitation, while others are experiencing greater decreases in precipitation (Dai, 2013; Easterling et al., 2000; Myhre et al., 2019). A meta‐analysis showed that increased precipitation did not significantly affect performance‐related traits (e.g., survival, growth, and reproduction) of both non‐native and native plant species, while decreased precipitation significantly inhibited these traits, with a slightly stronger negative impact on non‐natives than on natives (Liu et al., 2017). In other words, lower water availability is more stressful for non‐natives than for natives (Lucero et al., 2022;Valliere et al., 2019; Zhang et al., 2022). Furthermore, observational and modeling studies illustrate that climate warming affects not only the overall precipitation level but also patterns of precipitation (e.g., temporal separation and intensity of precipitation events), resulting in extensive fluctuations in available water for plants (Myhre et al., 2019). Fluctuation in water availability has a notable effect on plant growth and reproduction (Didiano et al., 2016, 2018; Sher et al., 2004), community composition (Shaw et al., 2022), and ecosystem productivity (Gherardi & Sala, 2019; Zhang, Biederman, et al., 2021). Thus, it is reasonable to assume that fluctuations in water availability will inevitably impact non‐native plants, but experimental evidence is limited if such impacts differ from those on native plants.

Fluctuation in water availability is characterized by repeated wetting–drying cycles. As mentioned above, non‐native plants are generally less tolerant to drought than natives (Valliere et al., 2019; Zhang et al., 2022). In contrast, non‐native plants benefit more from rewetting after drought (Diez et al., 2012). For example, Zhang et al. (2022) compared the growth of four pairs of congeneric non‐native and native plant species in response to rewetting after drought and showed that rewetting significantly increased plant biomass, with non‐natives displaying a stronger increase than for natives. Leal et al. (2022) also found similar results that non‐native plant species had greater recovery to drought than natives. As such, the effect of fluctuation in water availability on non‐native plants may depend on the net effects of tolerance and recovery to drought. Furthermore, drought tolerance and recovery are often influenced by drought duration and rewetting intensity (Bottero et al., 2021; Kelso et al., 2020; Xu et al., 2021). Together, the impact of fluctuation in water availability (wetting‐drying cycles) on non‐native plants is probably more complex than the impact of changes in amount of water availability (low vs. high), yet whether fluctuation in water availability has different impact on non‐native versus native species is largely unknown.

**FIGURE 1:**
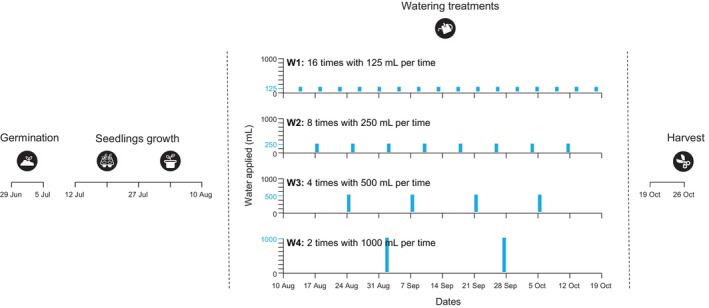
Graphical illustration of the experimental set‐up. We used seven non‐native and native species and grew them over a 17‐week period. We applied four watering treatments (W1–W4) differing in watering frequency, but with equivalent amounts of total water addition (2000 mL). Please see details in the Material and Methods section. Information of species and dates of water addition are given in Tables S1 and S2, respectively.

In this study, we conducted a pot experiment to test the effect of fluctuation in water availability on the responses of seven native and seven non‐native plant species. We exposed plants individually to four watering treatments with different frequency of water supply but held the total water supply constant (Figure 1). Thus, for these treatments, the watering intervals gradually increased along with the size of the watering pulse. Biomass production is generally influenced by water stress (Jackson et al., 2024; Wilschut et al., 2022), and its re‐allocation from shoot to root is often reported as a plastic response for mitigating water stress by increasing the uptake of limiting water (Eziz et al., 2017; Poorter et al., 2012; Qi et al., 2019). For this, we harvested above‐ and belowground parts of each plant separately at the end of the watering treatments. Specifically, we asked the following three questions: (1) How does fluctuation in water availability impact plant biomass production and allocation? (2) Does the impact of fluctuation in water availability depend on the frequency of water supply (large infrequent high‐water supply vs. small frequent low‐water supply)? (3) Are responses to fluctuation in water availability different for native and non‐native plant species?

## 2 MATERIALS AND METHODS

### 2.1 Study species

We selected seven non‐native and seven native plant species, which are common around farmlands and along roadsides in central China (Table S1). They belong to Amaranthaceae, Compositae, Fabaceae, Gramineae, and Lamiaceae families across three functional groups. Non‐native species included four herbs (*Ambrosia artemisiifolia*, *Bidens alba*, *Bidens frondosa*, and *Celosia argentea*), two grasses (*Paspalum urvillei* and *Paspalum wettsteinii*), and one legume (*Sesbania cannabina*). Native species included four herbs (*Bidens parviflora*, *Chrysanthemum indicum*, *Leonurus artemisia*, and *Nepeta cataria*), one grass (*Digitaria sanguinalis*), and two legumes (*Aeschynomene indica* and *Cassia tora*). We selected these non‐native and native species mainly based on their relative abundance in the field, without considering their phylogenetic relatedness, because matching congeneric pairs of native and non‐native species is not always possible. For instance, it is impossible to select an *Ambrosia* species native to China matching for the invasive *Ambrosia artemisiifolia* used in this study because all species in genus *Ambrosia* are native to America. Furthermore, there is only one species in the genus *Sesbania* native to China (*Sesbania grandiflora*), but this species is not widely distributed in central China. However, species selection was done to assure that species composition regarding different functional groups was similar between non‐native and native origins. We collected seeds of all species from field around Wuhan Botanical Garden (WBG), Chinese Academy of Sciences, Wuhan, China (30.51° N, 114.54° E) and stored them separately at 4°C until germination.

### 2.2 Experimental set‐up

To investigate the effect of fluctuation in water availability on the growth of non‐native and native plant species, we performed a pot experiment in a greenhouse with 28/20°C and 14/10 hours day/night cycle at WBG in 2020. We sowed the seeds of different species in an individual plastic box (16 × 16 × 7 cm) filled with growth substrate (Pindstrup Plus‐Orange Mosebrug, Burgos, Spain) over a week because of their inconsistent germination times (Table S1; Figure 1, 29 June–5 July). One week after the last species was sowed, we transplanted seedlings individually into 50‐cell seeding trays filled with same growth substrate (Figure 1, 12 July). Then, we selected similar‐sized seedlings of all species and transplanted them individually into 1.5 L round plastic pots (Figure 1, 27 July). The pots were filled with a mixture of field soil, sand, and vermiculite (v/v/v, 1:1:1). Field soil was collected from the top 15 cm at three locations around WBG, and sand and vermiculite were purchased from commercial vendors (Green Hope, Shenzhen, China). We added 80 mL of 400% strength Hoagland solution to each pot once per week for 2 weeks and watered it with 125 mL of water every 2–3 days (Figure 1, 27 July–10 August). Then, we started watering treatments for 10 weeks (Figure 1, 10 August–19 October).

We applied four treatments (W1–W4) differing in watering frequencies, but had equivalent amounts of total water addition (Table S2, Figure 1). Thus, treatments with higher watering frequencies would have less water addition each time. Based on precipitation datasets, a duration of <4 days between precipitation events (≥2 mm) was found to be dominant during the growing season (April to August) from 2000 to 2019 in Wuhan, accounting for 56.7% of all events (http://tjj.hubei.gov.cn, Figure S1). Thus, for W1, we added water 16 times with 125 mL per time for each pot, resulting in an addition interval of 3–4 days (Table S2). Given that more heavy precipitation events and longer dry periods are projected to increase over large parts of the world (Masson‐Delmotte et al., 2021), we proportionally increased the amount of water for a single watering addition and decreased the frequency of watering for W2–W4. Specifically, for W2, we added water eight times with 250 mL per time for each pot (Table S2). For W3, we added water four times with 500 mL per time for each pot (Table S2). For W4, we added water twice with 1000 mL per time for each pot (Table S2). To ensure that water did not outflow from the base of the pot in W4, we added two separate additions of each 1000 mL water within one day. There were 12 replicates for each species and watering treatment combination, resulting in 672 pots (2 origins × 7 species per origin × 4 watering treatments × 12 replicates). During the watering treatments, we used a TDR 100 soil moisture meter (Time Domain Reflectometer, Spectrum Technologies, Aurora, IL, USA) to measure volumetric water content (VWC) 33, 27, 20, and 15 times for W1, W2, W3, and W4, respectively. We also randomized the positions of pots every 2 weeks to reduce the impact of environmental heterogeneity in the greenhouse. One week after the end of the watering treatments (Figure 1, 26 October), we harvested above‐ and belowground parts of each plant, dried them separately at 60°C for 72 h, and weighed.

### 2.3 Data analysis

We calculated the time‐averaged weekly VWCs and the temporal variability of VWCs (coefficient of variation, CV) for each watering treatment. To examine the effect of watering treatment (W1, W2, W3, or W4) on average VWC and CV of VWC at the end of experiment, we used linear models (LMs). We performed multiple comparisons using least squared mean post hoc tests (LSM) and corrected P‐values using false discovery rate (FDR) (Benjamini & Hochberg, 1995).

To test variations in total biomass (belowground biomass + aboveground biomass) and root‐to‐shoot ratio (belowground biomass/aboveground biomass) at the end of the experiment, we used linear mixed models (LMMs). We considered plant origin (non‐native vs. native) and watering treatment (W1, W2, W3, or W4) as fixed effects and species (seven species per origin) nested within origin as random effects. We performed multiple comparisons for each plant origin and corrected P‐values as described above. We used LMs to test the effect of watering treatment on total biomass and root‐to‐shoot ratio for each species, followed by LSM post hoc tests and *p*‐value corrections.

To improve the normality of residuals, square‐root and log transformations were applied to total biomass and root‐to‐shoot ratio, respectively. All statistical analyses were carried out with R ver. 4.2.1 (https://www.r‐project.org) using “lme4,” “car,” “emmeans,” and “multcomp” packages (Bates et al., 2015; Fox & Weisberg, 2018; Hothorn et al., 2008; Lenth, 2021).

## 3 RESULTS

**FIGURE 2:**
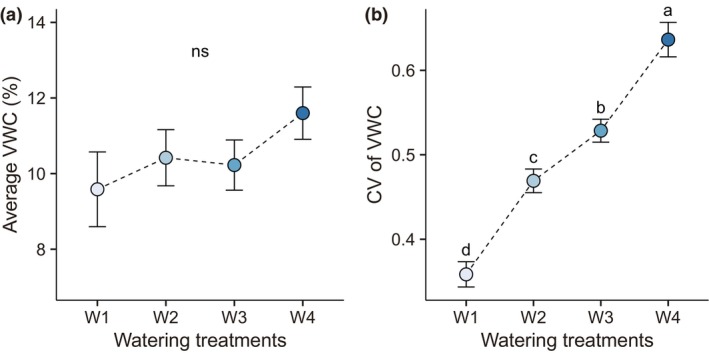
Effect of watering treatments on average VWC (a) and CV of VWC (b). We applied four treatments (W1, white; W2, light blue; W3, blue, and W4, dark blue), which were different in watering frequency, but had equivalent amounts of total water addition (see Figure 1 for details). Means ±1 SE are shown. Significant differences among watering treatments are indicated by different letters (*p* < .05). “ns” represents no significant difference between treatments.

There was no significant difference in the average VWC among the watering treatments (*F*_3,276_ = 1.16, *p* = .327, Figure 2a). However, the CV of VWC was different among the watering treatments (*F*_3,276_ = 52.64, *p* < .001, Figure 2b). The smallest fluctuation was observed for the many/small (W1) watering treatment, while the largest fluctuation was observed for the few/large (W4) watering treatment (Figure 2b, Figure S2).

**FIGURE 3:**
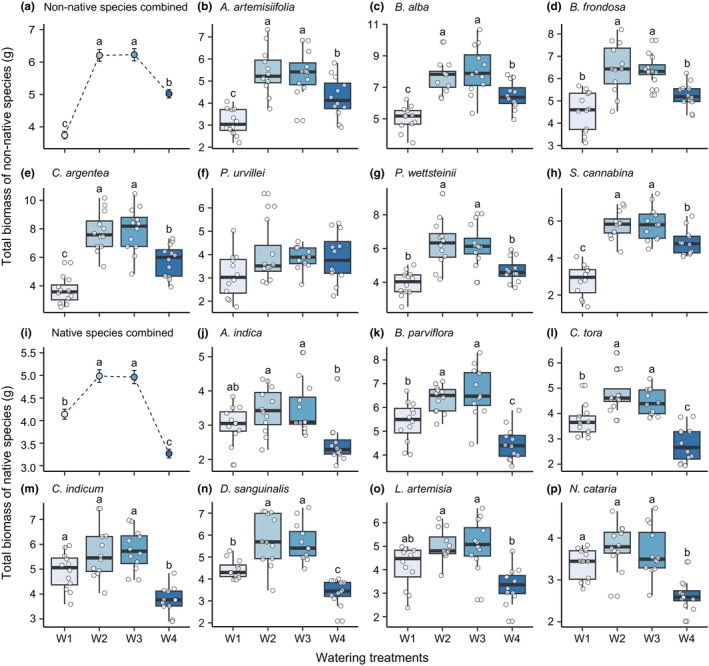
Impact of fluctuation in water availability on total biomass. We applied four treatments (W1, white; W2, light blue; W3, blue, and W4, dark blue), which were different in watering frequency, but had equivalent amounts of total water addition (see Figure 1 for details). Effects of watering treatments on total biomass of all non‐native species combined (*n* = 84, a) and on total biomass of each non‐native species (*n* = 12, b–h). Effects of watering treatments on total biomass of all native species combined (*n* = 84, i) and on total biomass of each native species (*n* = 12, j–p). Species information is in Table S1. The points in (a) and (i) represent the mean values of the treatments, and the error bars extending from each point represent the standard errors (SE). The boxes in (b–h and j–p) represent the interquartile range (IQR), with the whiskers extending to the minimum and maximum values within 1.5 times the IQR from the box. Different letters indicate significant differences (*p* < .05) among watering treatments in post hoc multiple comparisons.

Total biomass was significantly affected by watering treatment (*χ* ^2^ = 499.61, *p* < .001), plant origin (*χ* ^2^ = 3.01, *p* = .08), and their interaction (*χ* ^2^ = 154.49, *p* < .001). Non‐native and native plants produced the largest biomass in medium frequency/magnitude (W2 and W3) watering treatments (Figure 3a,i). However, non‐native plants had the lowest biomass in many/small watering treatment (Figure 3a), while native plants had the lowest biomass in few/large watering treatment (Figure 3i). Similar results were found for most non‐native and native species (Figure 3b–h, j–p, Table S3).

**FIGURE 4:**
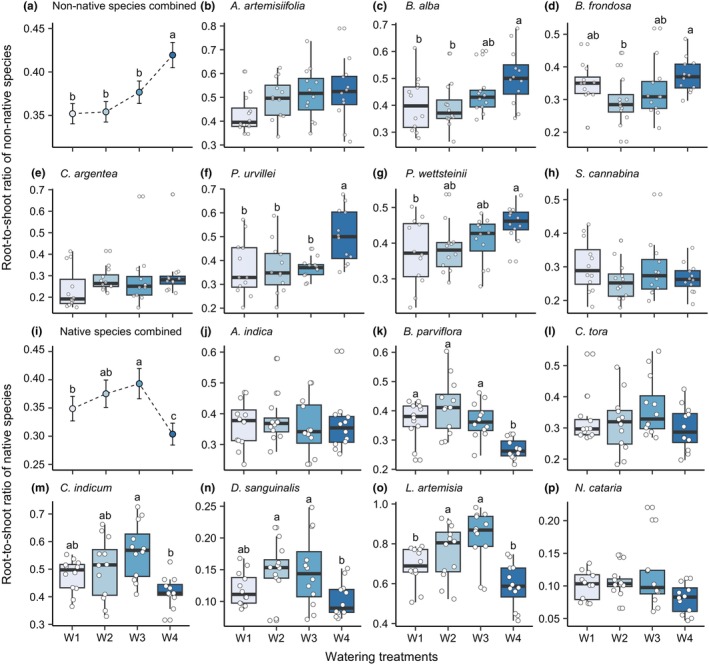
Impact of fluctuation of water availability on root‐to‐shoot ratio. We applied four treatments (W1, white; W2, light blue; W3, blue, and W4, dark blue), which were different in watering frequency, but had equivalent amounts of total water addition (see Figure 1 for details). Effects of watering treatments on root‐to‐shoot ratio of all non‐native species combined (*n* = 84, a), and on root‐to‐shoot ratio of each non‐native species (*n* = 12, b–h). Effects of watering treatments on root‐to‐shoot ratio of all native species combined (*n* = 84, i) and on root‐to‐shoot ratio of each native species (*n* = 12, j–p). Species information is in Table S1. The points in (a) and (i) represent the mean values of the treatments, and the error bars extending from each point represent the standard errors (SE). The boxes in (b–h and j–p) represent the interquartile range (IQR), with the whiskers extending to the minimum and maximum values within 1.5 times the IQR from the box. Different letters indicate significant differences (*p* < .05) among watering treatments in post hoc multiple comparisons.

Root‐to‐shoot ratio was not affected by plant origin (*χ* ^2^ = 0.62, *p* = .433), but strongly responded to watering treatment (*χ* ^2^ = 12.13, *p* = .007) and its interaction with plant origin (*χ* ^2^ = 72.08, *p* < .001). Under many/small and few/large watering treatments, non‐native and native plants exhibited opposite trends in root‐to‐shoot ratio. Non‐native plants had a higher root‐to‐shoot ratio under few/large watering treatment (Figure 4a), while native plants had a lower ratio under many/small watering treatment (Figure 4i). Similar root‐to‐shoot ratio response patterns were found for most of non‐native and native species (Figure 4b–h, j–p, Table S3).

## 4 DISCUSSION

Many studies document that changes in water availability strongly affect the growth of non‐native plants (Ali & Bucher, 2022; LaForgia et al., 2018; Liu et al., 2017; Valliere et al., 2019). Previous studies have primarily focused on the effect of variation in total amount of water availability (dry vs. wet), while ignoring the effect of fluctuation in water availability, although prolonged drought and extreme precipitation substantially increase due to climate change (Dai, 2013; Masson‐Delmotte et al., 2021; Thackeray et al., 2022). We examined the response of seven non‐native and seven native plant species to four watering treatments differing in watering frequency but had equivalent amounts of total water addition. We found that fluctuations in water availability strongly affected biomass production of non‐native and native plant species, while responses to extreme low and high fluctuations were different between them. Non‐native plants relatively grew better with infrequent, substantial watering treatment, whereas native plants relatively favored frequent, minor watering treatment.

We found that plants produced more biomass under medium frequency/magnitude watering treatments (W2, eight times with 250 mL per time; W3, four times with 500 mL per time), while produced less biomass under many/small (W1, 16 times with 125 mL per time) and few/large (W4, two times with 1000 mL per time) watering treatments (Figure 3). This pattern was observed for both non‐native and native species and is consistent with previous studies that applied different frequencies of watering additions but held total amounts of water addition constant. For instance, Gao et al. (2015) found that the annual plant *Agriophyllum squarrosum* generally produced less biomass and seeds under lower and higher watering frequencies than medium watering frequency. Zhang, Shen, et al. (2021) showed similar results in biomass and height for *Leymus chinensis*. In our study, many/small watering treatment (W1) caused a gradual decrease in VWC, resulting in a prolonged drought that occurred earlier than medium frequency/magnitude watering treatments (W2 and W3) (Figure S2a–c). In contrast, few/large watering treatment (W4) caused fluctuation in VWC, leading to periodic drought that occurred more pronounced than medium frequency/magnitude watering treatments (W2 and W3) (Figure S2b–d). Previous studies have shown that both prolonged drought and periodic drought are detrimental to plant growth, reproduction, and fitness (Knapp et al., 2023; Reyer et al., 2013; Volaire, 2018). Thus, the lower biomass production under the many/small (W1) and few/large (W4) watering treatments can most probably be attributed to early prolonged drought and extreme periodic drought, respectively.

Interestingly, although many/small (W1) and few/large (W4) watering treatments reduced plant growth compared to medium frequency/magnitude watering treatments (W2 and W3), non‐native and native plants exhibited opposite patterns under these two conditions (Figure 3). Generally, non‐native plants grew relatively better in few/large than in many/small watering treatments, while native plants grew relatively better in many/small than in few/large watering treatments. These different responses of non‐native and native plants may be attributed to their different drought tolerance as well as their ability to recover following rewetting events. Previous studies have shown that non‐native plants are less tolerant to drought than native plants (Liu et al., 2017; Oram et al., 2023; Valliere et al., 2019). In contrast, non‐native plants can take more advantages of rewetting events to recover than native plants (Zhang et al., 2022), whereas some native plants cannot recover or even experience waterlogging stress caused by rewetting events (Blom & Voesenek, 1996; Reyer et al., 2013). Therefore, non‐native plants produced less biomass under the many/small treatment than that under the few/large treatment, while native plants showed the opposite pattern.

Biomass allocation is an important mechanism to cope with resource stress for plants (Poorter et al., 2012; Qi et al., 2019). Optimal partitioning hypothesis suggests that plants tend to allocate more biomass to the organ that obtains the most limited resource (Bloom et al., 1985). Generally, among plant organs, roots are accountable for nutrient and water uptake, while leaves are primarily responsible for photosynthesis (Freschet et al., 2018; Hodge, 2009). Therefore, to cope with drought stress, plants will shift biomass partitioning toward less shoot production and more root production, resulting in a higher root‐to‐shoot ratio (Eziz et al., 2017; Zhou et al., 2018), which enhances water uptake by increasing root extension into deeper soil layers and/or increasing root surface area (Lozano et al., 2020; Reinelt et al., 2023). Consistent with the optimal partitioning hypothesis, we found that native plants exhibited higher root‐to‐shoot ratio in the many/small watering treatment than in the few/large watering treatments, while non‐native plants exhibited higher root‐to‐shoot ratio in the few/large watering treatment than in the many/small watering treatment (Figure 4), which may reflect the generally higher phenotypic plasticity of non‐native plants and/or their better adaptation to disturbed habitats with more prominent water and resource fluctuations as compared with native plants (Gentili et al., 2021; Hansen & Clevenger, 2005; Richards et al., 2006). Thus, the different pattern of root‐to‐shoot ratio in these two watering treatments between non‐native and native species may contribute to the observed different pattern of non‐native and native species biomass production. However, the root‐to‐shoot ratio of plants under both many/small and few/large watering treatments was lower than those under the medium frequency/magnitude watering treatments, which reflects the intricate response of the root‐to‐shoot ratio to the fluctuation in water availability in plants. Such complexity might be influenced by other factors, such as the total water amount received or the species specificity of the tested species (Gao et al., 2015; Padilla et al., 2013; Zhang, Shen, et al., 2021).

An important caveat is that we only included a limited number of non‐native and native species and did not consider the phylogenetic relatedness between them. Previous studies indicate that matching the phylogenetic relatedness between non‐native and native species is a powerful method for identifying potential invasive mechanisms (Engelkes et al., 2016; Manrubia et al., 2020; Yu et al., 2022). Because closely related plant species generally have similar ecological niche and environmental adaptation (Burns & Strauss, 2011; Davies et al., 2013). Thus, our species selection is a limitation, and further comparison of multiple phylogenetically controlled pairs of non‐native and native species is needed. Additionally, our tested species included grasses, herbs, and legumes with generally different growth rates (Reich et al., 2003; Wang & Tang, 2019), but our species selection was done to assure that species composition regarding different functional groups was similar between non‐native and native origins. Thus, our experiment with a duration of 17 weeks may be insufficient to fully reflect their responses to fluctuation of water availability, and future research should ideally incorporate long‐term manipulation experiments or engage in long‐term field observations, accounting for a similar composition of functional groups between the two origins.

In conclusion, our results indicate that the biomass production of both non‐native and native plants used in this study is influenced by the fluctuation in water availability. More importantly, non‐native plants performed better in the few/large watering treatment, while native plants performed better in the many/small watering treatment. Furthermore, our finding holds significant implications for gaining a deeper understanding of the response of plants from different origins to fluctuation in water availability and provides leads for further investigating the invasion dynamics under changes in precipitation patterns.

## AUTHOR CONTRIBUTIONS

**Wenchao Qin:** Conceptualization (equal); data curation (equal); investigation (lead); methodology (equal); writing – original draft (lead); writing – review and editing (equal). **Yan Sun:** Writing – review and editing (equal). **Heinz Müller‐Schärer:** Writing – review and editing (equal). **Wei Huang:** Conceptualization (equal); formal analysis (equal); funding acquisition (lead); writing – original draft (equal); writing – review and editing (lead).

## Supporting information


Table S1.

## Data Availability

The raw data associated with this study is publicly accessible in the Science Data Bank (https://doi.org/10.57760/sciencedb.08928).
