# Supplementary material for: Responses of non‐native and native plant species to fluctuations of water availability in a greenhouse experiment
Source: Ecol Evol. 2024 Jul 9;14(7):e11692. doi: 10.1002/ece3.11692 (PMC11232050; doi:10.1002/ece3.11692)

**Supporting information**

**Responses of non-native** **and native plant species to fluctuations of water availability in a greenhouse experiment**

Wenchao Qin^1,2^, Yan Sun^3^, Heinz Müller-Schärer^3,4^, Wei Huang^1,5*^

^1^ Wuhan Botanical Garden, Chinese Academy of Sciences, Wuhan 430074, China

^2^ University of Chinese Academy of Sciences, Beijing 100049, China

^3^ College of Resources and Environment, Huazhong Agricultural University, Wuhan 430070, China

^4^ Department of Biology, University of Fribourg, Fribourg 1700, Switzerland

^5^ Hubei Key Laboratory of Wetland Evolution & Ecological Restoration, Wuhan Botanical Garden, Chinese Academy of Sciences, Wuhan 430074, China

^*^ Corresponding author:

Wei Huang, [huangwei0519@wbgcas.cn](mailto:huangwei0519@wbgcas.cn) (ORCID iD: 0000-0003-3760-6084)

Tables: S1-S3

Figures: S1-S2

**Table S1.** Information of seven non-native plant species and seven native plant species used in the study.

| **Species** | **Species status** | **Native range** | **Family** | **Functional group** | **Life history** | **Germination date** |
| --- | --- | --- | --- | --- | --- | --- |
| *Ambrosia artemisiifolia* | Non-native | Central and North America | Compositae | Herb | Annual | 7/2/2020 |
| *Bidens alba* | Non-native | Tropical America | Compositae | Herb | Annual | 7/1/2020 |
| *Bidens frondosa* | Non-native | North America | Compositae | Herb | Annual | 7/1/2020 |
| *Celosia argentea* | Non-native | India | Amaranthaceae | Herb | Annual | 7/1/2020 |
| *Paspalum urvillei* | Non-native | South America | Gramineae | Grass | Perennial | 6/29/2020 |
| *Paspalum wettsteinii* | Non-native | South America | Gramineae | Grass | Perennial | 6/29/2020 |
| *Sesbania cannabina* | Non-native | Oceania to the Pacific Islands | Fabaceae | Legume | Annual | 7/3/2020 |
| *Aeschynomene indica* | Native |  | Fabaceae | Legume | Annual | 7/3/2020 |
| *Bidens parviflora* | Native |  | Compositae | Herb | Annual | 7/1/2020 |
| *Cassia tora* | Native |  | Fabaceae | Legume | Annual | 7/3/2020 |
| *Chrysanthemum indicum* | Native |  | Compositae | Herb | Perennial | 7/4/2020 |
| *Digitaria sanguinalis* | Native |  | Gramineae | Grass | Annual | 7/5/2020 |
| *Leonurus artemisia* | Native |  | Lamiaceae | Herb | Annual / Biennial | 7/4/2020 |
| *Nepeta cataria* | Native |  | Lamiaceae | Herb | Perennial | 6/29/2020 |

**Table S2.** Dates of water additions for the watering treatments, each treatment with the same total amount of water, but with different divisions. We applied four watering treatments (W1-W4) from 10 August to 19 October. For W1, we added water 16 times with an addition interval of 3-4 days. For W2, we added water eight times with an addition interval of 6-7 days. For W3, we added water four times with an addition interval of 14 days. For W4, we added water two times with an addition interval of 24 days.

| Treatments | Date |
| --- | --- |
| W1 | August 14, 2020 |
|  | August 18, 2020 |
|  | August 22, 2020 |
|  | August 27, 2020 |
|  | August 31, 2020 |
|  | September 5, 2020 |
|  | September 9, 2020 |
|  | September 14, 2020 |
|  | September 18, 2020 |
|  | September 23, 2020 |
|  | September 27, 2020 |
|  | October 1, 2020 |
|  | October 5, 2020 |
|  | October 10, 2020 |
|  | October 14, 2020 |
|  | October 18, 2020 |
| W2 | August 17, 2020 |
|  | August 25, 2020 |
|  | September 2, 2020 |
|  | September 10, 2020 |
|  | September 18, 2020 |
|  | September 26, 2020 |
|  | October 4, 2020 |
|  | October 11, 2020 |
| W3 | August 24, 2020 |
|  | September 7, 2020 |
|  | September 21, 2020 |
|  | October 5, 2020 |
| W4 | September 2, 2020 |
|  | September 27, 2020 |

**Table S3.** The results of LMs for the effects of watering treatment on total biomass and root-to-shoot ratio for each species. Significant effects (*P* < 0.05) are bolded, and marginally significant effects (0.05 < *P* <0.1) are underlined.

|  | **Total biomass (g)** | |  | **Root-to-shoot ratio (%)** | |
| --- | --- | --- | --- | --- | --- |
|  | **F_3, 44_** | **P** |  | **F_3, 44_** | **P** |
| **Non-native species** |  |  |  |  |  |
| *Ambrosia artemisiifolia* | 16.90 | **<0.0001** |  | 2.20 | 0.102 |
| *Bidens alba* | 19.41 | **<0.0001** |  | 3.52 | **0.023** |
| *Bidens frondosa* | 14.77 | **<0.0001** |  | 2.65 | 0.060 |
| *Celosia argentea* | 28.78 | **<0.0001** |  | 0.87 | 0.465 |
| *Paspalum urvillei* | 2.08 | 0.116 |  | 5.41 | **0.003** |
| *Paspalum wettsteinii* | 14.81 | **<0.0001** |  | 3.22 | **0.032** |
| *Sesbania cannabina* | 36.87 | **<0.0001** |  | 1.12 | 0.351 |
| **Native species** |  |  |  |  |  |
| *Aeschynomene indica* | 6.00 | **0.002** |  | 0.19 | 0.901 |
| *Bidens parviflora* | 17.93 | **<0.0001** |  | 9.83 | **<0.0001** |
| *Cassia tora* | 22.70 | **<0.0001** |  | 1.37 | 0.264 |
| *Chrysanthemum indicum* | 15.59 | **<0.0001** |  | 5.91 | **0.002** |
| *Digitaria sanguinalis* | 23.24 | **<0.0001** |  | 3.73 | **0.018** |
| *Leonurus artemisia* | 10.92 | **<0.0001** |  | 8.88 | **<0.0001** |
| *Nepeta cataria* | 14.50 | **<0.0001** |  | 2.50 | 0.072 |

**Figure S1.** Distribution of precipitation event intervals during the growing seasons (April to August) from 2000 to 2019 in Wuhan, China. The intervals of precipitation events lasting less than 4 days were the most common, accounting for 56.7%. The longest precipitation interval ranged between 29-32 days, representing only 0.2%. Precipitation data was extracted from Hubei Provincial Statistic Bureau (<http://tjj.hubei.gov.cn>).


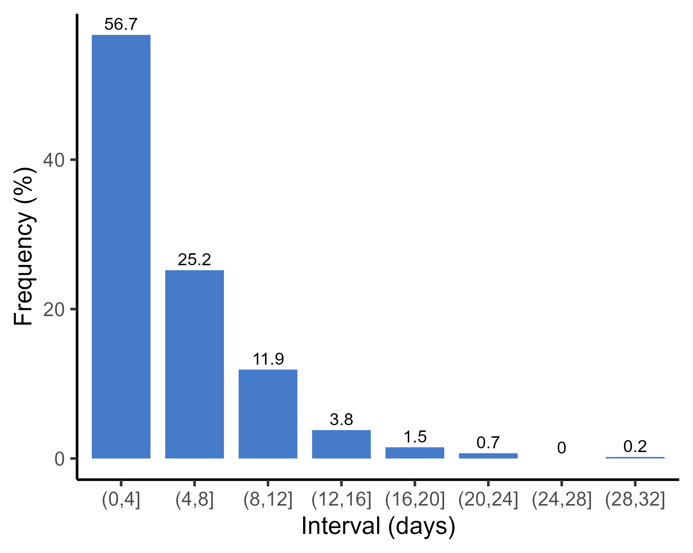


**Figure S2.** Mean soil volumetric water content (VWC) during the period of watering treatments. We applied four watering treatments (W1-W4), which were different in watering frequency, but had equivalent amounts of total water addition. We measured VWC 33, 27, 20 and 15 times for W1 (a), W2 (b), W3 (c) and W4 (d), respectively.


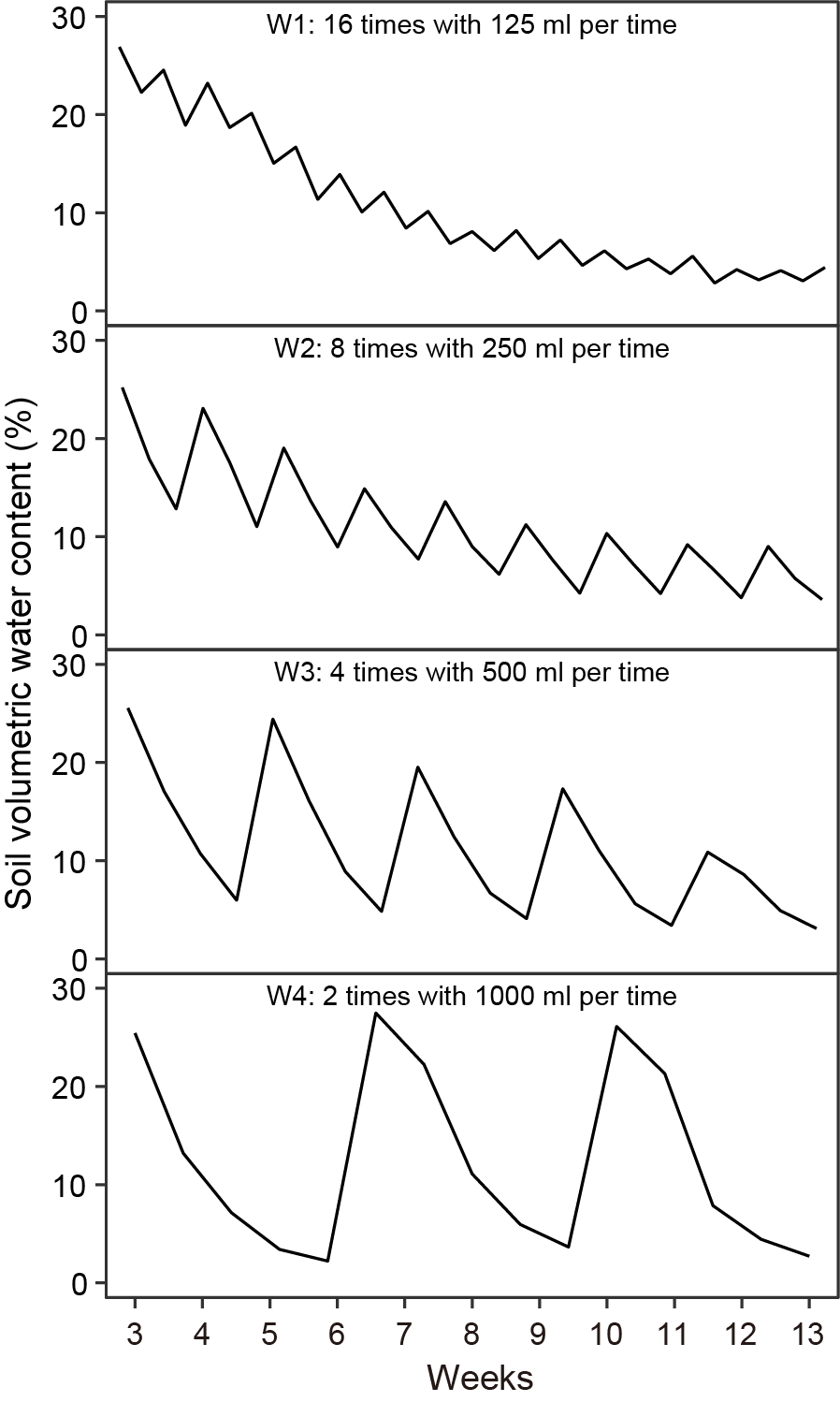

Supplement: Supplementary file 1 — Table S1. [file ECE3-14-e11692-s001.docx]
